# Raw pacific biosciences and illumina sequencing reads and assembled genome data for the cattle ticks *Rhipicephalus microplus* and *Rhipicephalus annulatus*

**DOI:** 10.1016/j.dib.2021.106852

**Published:** 2021-02-06

**Authors:** Felix D. Guerrero, Noushin Ghaffari, Kylie G. Bendele, Richard P. Metz, C. Michael Dickens, Philip D. Blood, Jason Tidwell, Robert J. Miller, Adalberto A. Pérez de León, Pete D. Teel, Charles D. Johnson

**Affiliations:** a USDA-ARS Knipling-Bushland US Livestock Insect Research Laboratory and Veterinary Pest Genomics Center, 2700 Fredericksburg Road, Kerrville, TX 78028, USA; b Department of Computer Science, Roy G. Perry College of Engineering, Prairie View A&M University, Prairie View, TX 77446, USA; c Genomics and Bioinformatics Service, Texas A&M AgriLife Research, 101 Gateway, Suite A, Room 121, College Station, TX 77845, USA; d Texas A&M High Performance Research Computing, Texas A&M University, 1500 Research Parkway, Suite 250, 1157B Interdisciplinary Life Sciences Building, College Station, TX 77845, USA; e Pittsburgh Supercomputing Center, Carnegie Mellon University, Pittsburgh, PA 15213, USA; f USDA-ARS Cattle Fever Tick Research Laboratory, 22675 Moorefield Road, Edinburg, TX 78541, USA; g Department of Entomology, Texas A&M University, College Station, TX 77845, USA

**Keywords:** Rhipicephalus microplus, Rhipicephalus annulatus, PacBio genome sequencing, Large genome assembly, Canu assembler, Cattle tick

## Abstract

Ticks from the genus *Rhipicephalus* have enormous global economic impact as ectoparasites of cattle. *Rhipicephalus microplus* and *Rhipicephalus annulatus* are known to harbor infectious pathogens such as *Babesia bovis, Babesia bigemina*, and *Anaplasma marginale*. Having reference quality genomes of these ticks would advance research to identify druggable targets for chemical entities with acaricidal activity and refine anti-tick vaccine approaches. We sequenced and assembled the genomes of *R. microplus* and *R. annulatus*, using Pacific Biosciences and HiSeq 4000 technologies on very high molecular weight genomic DNA. We used 22 and 29 SMRT cells on the Pacific Biosciences Sequel for *R. microplus* and *R. annulatus*, respectively, and 3 lanes of the Illumina HiSeq 4000 platform for each tick. The PacBio sequence yields for *R. microplus* and *R. annulatus* were 21.0 and 27.9 million subreads, respectively, which were assembled with Canu v. 1.7. The final Canu assemblies consisted of 92,167 and 57,796 contigs with an average contig length of 39,249 and 69,055 bp for *R. microplus* and *R. annulatus*, respectively. Annotated genome quality was assessed by BUSCO analysis to provide quantitative measures for each assembled genome. Over 82% and 92% of the 1066 member BUSCO gene set was found in the assembled genomes of *R. microplus* and *R. annulatus*, respectively. For *R. microplus*, only 189 of the 1066 BUSCO genes were missing and only 140 were present in a fragmented condition. For *R. annulatus*, only 75 of the BUSCO genes were missing and only 109 were present in a fragmented condition. The raw sequencing reads and the assembled contigs/scaffolds are archived at the National Center for Biotechnology Information.

## Specifications Table

**Table:** 

Subject	Biology
Specific subject area	Genomics
Type of data	Assembled genome sequences and tables displaying sequencing, assembly, and repeats analysis statistics
How data were acquired	Long-read sequencing of very high molecular weight genomic DNA using Pacific Biosciences Sequel and Illumina HiSeq 4000
Data format	Pacific Biosciences raw data in bam format, Illumina HiSeq 4000 raw data in fastq format
	CANU-assembled Pacific Biosciences-only contigs/scaffolds in fasta format
Parameters for data collection	The expected large genome size of these ticks necessitated the usage of long read sequencing technology and a genomic DNA isolation technique capable of purifying very high molecular weight DNA. Approximately 0.4 mg of this genomic DNA was sequenced to produce our genome assembly. The inbred nature of the laboratory sourced tick strains would reduce the heterogeneity of the genomic DNA, thus assisting the assembly of reads into contigs and scaffolds.
Description of data collection	Eggs collected from laboratory-reared adult females ticks were used to purify very high molecular weight genomic DNA, using a proteinase K/RNAse A/phenol-based extraction protocol. This DNA was sequenced on the Pacific Biosciences Sequel and Illumina HiSeq 4000 platforms. The Sequel reads were assembled using CANU, polished using ArrowGrid, and haplotigs separated from primary contigs by Purge_Haplotigs. The *R. microplus* genome assembly was further improved with chromosome conformation capture HiC analysis using Dovetail Genomic's proprietary algorithm HiRise. Genome completeness was assessed with Busco in genome analysis mode.
Data source location	United States Department of Agriculture, Agricultural Research Service Cattle Fever Tick Research Laboratory
	Edinburg, TX
	United States of America
	Latitude: 26.398596
	Longitude: -98.344652
	GPS: 26.398596, -98.344652
Data accessibility	Repository name:
	National Center for Biotechnology Information
	Direct URL to data:
	https://www.ncbi.nlm.nih.gov
	Data identification number:
	The sequence data for this project can be found at the National Center for Biotechnology Information (NCBI) under BioProject accession numbers PRJNA552342 (*R. microplus*) and PRJNA593711 (*R. annulatus*). The BioSample accession numbers for the *R. microplus* sample is SAMN06075429 and the *R. annulatus* sample is SAMN12497614. The raw read data can be found in the Sequence Read Archive (SRA) under accession numbers SRR9875273 for the *R. microplus* PacBio Sequel reads, SRR10034978 for the *R. microplus* Illumina Dovetail Hi-C reads and SRR10009121 for the *R. annulatus* PacBio Sequel reads, SRR12508557-SRR12508563 for the *R.annulatus* Illumina HiSeq reads, and SRR13614643-SRR13614649 for the *R. microplus* Illumina HiSeq reads. The assembled genomes can be found under Whole Genome Shotgun (WGS) accession numbers WOVZ00000000 for *R. microplus* and WOVY00000000 for *R. annulatus*.
	https://www.ncbi.nlm.nih.gov/sra/?term=SRR10009121
	https://www.ncbi.nlm.nih.gov/sra/?term=SRR10034978
	https://www.ncbi.nlm.nih.gov/sra/?term=SRR9875273

## Value of the Data

• These are high quality genomes of important cattle parasites that vector bovine pathogens.
• Researchers studying arachnid and tick genomics, comparative genomics, and arachnid evolution will find the assembled genomes valuable.
• The datasets can be used to study genes involved in the development of pesticide resistance in these economically important tick species.
• Genes present in these genomes can provide foundational data for research to identify druggable targets for chemical entities with acaricidal activity and also refine anti-tick vaccine approaches.

## 1 Data Description

**Fig. 1:**
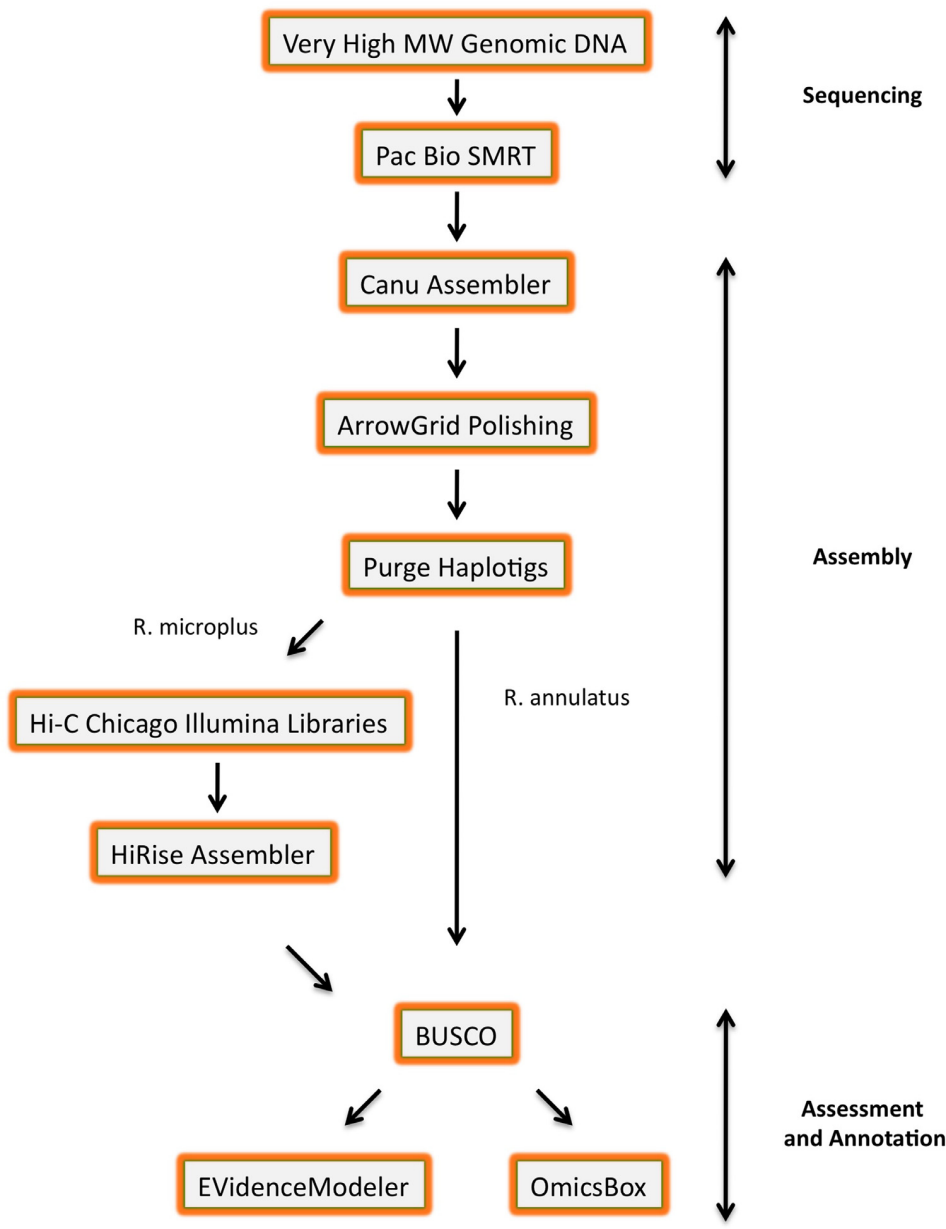
Flow diagram of the genome sequencing, assembly, quality assessment, and annotation process.

*Rhipicephalus microplus* and *R. annulatus* are known to harbor infectious pathogens including *Babesia bovis, Babesia bigemina*, and *Anaplasma marginale*. Bovine babesiosis is considered the most economically important arthropod vector-borne disease of livestock in the world. *R. microplus* is also of high consequence to animal agriculture in tropical and subtropical parts of the world where it has developed resistance to all available commercial pesticide products [1]. Very high molecular weight genomic DNA was purified from eggs collected from laboratory-reared strains of *R. microplus* and *R. annulatus*. The genomic DNA was sequenced using 22 and 29 SMRT cells for *R. microplus* and *R. annulatus*, respectively, on Pacific Biosciences Sequel and 3 lanes on the Illumina HiSeq 4000 platform. The Canu assembler was used to assemble the genome using only the PacBio reads. Raw read data can be found in the Sequence Read Archive (SRA) under accession numbers SRR9875273 for the *R. microplus* PacBio Sequel reads, SRR10034978 for the *R. microplus* Illumina Dovetail Hi-C reads and SRR10009121 for the *R. annulatus* PacBio Sequel reads, SRR12508557-SRR12508563 for the *R.annulatus* Illumina HiSeq reads, and SRR13614643-SRR13614649 for the *R. microplus* Illumina HiSeq reads. The assembled genomes can be found under Whole Gun Shotgun (WGS) accession numbers WOVZ00000000 for *R. microplus* and WOVY00000000 for *R. annulatus*. The BioProject accession numbers are PRJNA552342 (*R. microplus*) and PRJNA593711 (*R. annulatus*). Information about the sequence reads, assembled genomes, and BUSCO analyses are presented in Tables 1, 2, and 3, respectively. Fig. 1 is a process flow diagram to clarify the data processing and genome assembly steps.

**Table 1:** Statistics of the *R. microplus* and *R. annulatus* sequence reads.

	*R. microplus*	*R. annulatus*
Total SMRT cells	22	29
Total Subreads^a^	21,012,044	27,870,925
Overall Subread Mean length	7,344 bp	6,527
Total bp	154,312,451,136	181,913,527,475
Genome coverage^b^	53 X	59 X
Subread N50	5,719 bp	5,393

a These are reads ultimately used in the genome assembly

b Based on estimated genome size of 2.90 and 3.06 Gb for *R. microplus* and *R. annulatus*, respectively.

**Table 2:** Statistical measures of the *R. microplus* and *R. annulatus* Canu assemblies, Arrow polished assemblies, and after Purge Haplotig.

					Purge Haplotigs			
	Canu		Arrow		Contigs		Haplotigs	
	Microplus	Annulatus	Microplus	Annulatus	Microplus	Annulatus	Microplus	Annulatus
Assumed genome size (Gbp)	2.90	3.06	2.90	3.06	2.90	3.06	2.90	3.06
Total contig length (% of assumed genome size)	124.7	130.4	125.0	130.6	82.7	90.3	40.9	37.9
Number of contigs	92,167	57,796	92,167	57,796	35,303	16,339	52,863	35,965
Number of contigs in scaffolds	0	0	0	0	0	0	0	0
Total size of contigs (Gbp)	3.6174	3.9911	3.6256	3.9957	2.3993	2.7624	1.1849	1.1591
Longest contig (bp)	1,581,913	5,211,643	1,582,208	5,213,396	1,582,208	5,213,396	806,872	766,744
Shortest contig (bp)	1,000	1,000	1,000	1,000	1,004	1,001	1,006	1,048
Number of contigs > 1K nt	92,166	57,795	92,166	57,795	35,303	16,339	52,863	35,965
Number of contigs > 10K nt	77,710	53,331	77,810	53,358	32,386	15,352	43,790	34,371
Number of contigs > 100K nt	7,050	7,594	7,073	7,603	6,779	6,470	291	1,122
Number of contigs > 1M nt	15	376	15	376	15	375	0	0
Number of contigs > 10M nt	0	0	0	0	0	0	0	0
Mean contig size (bp)	39,249	69,055	39,337	69,134	67,964	169,070	22,414	32,228
Median contig size (bp)	21,553	25,123	21,631	25,165	38,099	61,912	18,395	24,283
N50 contig length (bp)	67,334	236,157	67,400	236,076	121,485	436,999	27,206	36,113
L50 contig count	11,957	3,592	11,976	3,595	5,210	1,685	13,708	8,483
NG50 contig length (bp)	95,922	383,411	96,307	384,059	91,843	374,449	na	na
LG50 contig count	7,477	2,036	7,456	2,034	7,594	2,053	na	na
N50 contig - NG50 contig length difference (bp)	28,588	147,254	28,907	147,983	29,642	62,550	27,206	na
contig %A	27.17	27.09	27.19	27.1	27.18	27.14	27.22	27.03
contig %C	22.82	22.89	22.8	22.88	22.8	22.86	22.76	22.93
contig %G	22.82	22.88	22.81	22.88	22.82	22.86	22.77	22.9
contig %T	27.2	27.14	27.21	27.14	27.2	27.14	27.26	27.14
contig %N	0	0	0	0	0	0	0	0
contig %non-ACGTN	0	0	0	0	0	0	0	0
Number of contig non-ACGTN nt	0	0	0	0	0	0	0	0

**Table 3:** BUSCO statistics for the *R. microplus* and *R. annulatus* assembled genomes.

	Canu		Arrow		Purge Haplotigs	
	Microplus	Annulatus	Microplus	Annulatus	Microplus	Annulatus
Total BUSCO groups searched	1066	1066	1066	1066	1066	1066
Number of BUSCO complete and single copy (% of total)	526 (49.3%)	736 (69.0%)	690 (64.7%)	760 (71.3%)	829 (77.8%)	857 (80.4%)
Number of BUSCO complete and duplicated (% of total)	211 (19.8%)	146 (13.7%)	191 (17.9%)	119 (11.2%)	33 (3.1%)	31 (2.9%)
Number of BUSCO fragmented (% of total)	140 (13.1%)	109 (10.2%)	108 (10.1%)	113 (10.6%)	119 (11.2%)	113 (10.6%)
Number of BUSCO missing (% of total)	189 (17.8%)	75 (7.0%)	77 (7.2%)	74 (6.9%)	85 (8.0%)	65 (6.1%)

## 2 Experimental Design, Materials and Methods

### 2.1 Tick materials and genomic DNA purification

For *R. microplus*, genomic DNA was extracted from 10 g of a pooled collection of eggs obtained from the f7, f10, f11, and f12 generation of the Deutsch strain. The Deutsch strain was started from a few individual engorged female ticks collected during a 2001 tick outbreak in Webb County, TX, USA. For *R. annulatus*, we sought to reduce genetic heterozygosity by conducting single pair matings of generation 18 of the Klein Grass strain, placing one adult male with 10 female adults in a cloth sleeve glued to the shaved side of a bovine host. Following engorgement, individual females were placed into tubes to enable oviposition. We obtained a total of 1.25 g of eggs from 9 single paired matings and this amount of eggs yielded 1.7 mg of genomic DNA. The Klein Grass strain was started in 2010 from an outbreak in Kinney County, TX, USA. Both tick strains have been inbred since their collection and creation, however, they are not genetically homogeneous. A protocol from Sambrook et al. [2] was used to purify very high molecular weight genomic DNA, pulverizing frozen eggs in a liquid nitrogen-cooled mortar and pestle, addition to an aqueous buffer, followed by RNAse treatment, proteinase K digestion, phenol extraction, and dialysis in 50 mM Tris, 10 mM EDTA, pH 8.0 [3]. The resultant DNA was determined by agarose gel electrophoresis to be > 200 kb.

### 2.2 Genome sequencing and assembly

Sequencing at the Texas A&M AgriLife Genomics and Bioinformatics Service, College Station, TX used 22 and 29 SMRT cells on the Pacific Biosciences Sequel for *R. microplus* and *R. annulatus*, respectively. Each genomic DNA was also sequenced on 3 lanes of the Illumina HiSeq 4000 platform. The Illumina reads were originally intended for use in error-correcting the Sequel long reads. However, as we could not access the computational resources necessary to error-correct and assemble these large tick genomes, we chose to create a Sequel-only assembly using the Canu pipeline [4]. Read quality checks and filtering of raw reads were conducted via the manufacturer's standard protocol and protocols developed at the Texas A&M AgriLife Genomics and Bioinformatics Service prior to submission to NCBI and assembly. Canu software error corrects the long reads in multiple steps and can generate highly contiguous genome assembly. We utilized the Pittsburgh Supercomputing Center *Bridges* system [5], granted through the National Science Foundation-sponsored Extreme Science and Engineering Discovery Environment (XCEDE) program [6]. Each tick genome's Canu assembly took approximately 25 consecutive days, running on a reserved node with access to 352 cores, 12 TB of RAM, and node-local disk storage to avoid unnecessary data transfers. Program parameters were corMhapSensitivity=high, corOutCoverage=100, batOptions=-dg3 -db 3 -dr 1 -ca 500 -cp 50, and an input genome size estimate of 2.9 and 3.0 Gb for *R. microplus* and *R. annulatus*, respectively, based upon our studies with *Rhipicephalus* tick genomes (F. Guerrero, unpublished results).

Two rounds of polishing the assembly were performed using the ArrowGrid [7] wrapper tool, which incorporates the PacBio GenomicConsensus v2.3.2 Arrow algorithm. The ArrowGrid installation included ArrowGrid commit d3aa0f3 dated July 18, 2018, and the PacBio pbbioconda Github repository (https://github.com/PacificBiosciences/pbbioconda) commit 1d1dd31 dated September 25, 2018. The scheduler part of the ArrowGrid workflow tool was adapted to run on the Texas A&M High Performance Computing (HPRC) Terra cluster, which uses the Slurm Workload Manager. BamTools v2.5.1 (https://github.com/pezmaster31/bamtools) was also used in the ArrowGrid workflow. Purge_Haplotigs v1.0.4 [8] was used to separate primary contigs from haplotigs on the assembled contigs after the second round of Arrow polishing. Purge_Haplotigs was also used to generate the NCBI placement file, which provides genomic coordinates of the haplotigs relative to the primary contigs. NUCmer v3.1 with MUMmer 3.2.3 was used for each purge_haplotigs step and NUCmer v3.9.0alpha with MUMmer version 3.9.0alpha was used to generate the NCBI placement file since the purge_haplotigs ncbiplace command required NUCmer v3.9+.

In order to further improve the quality of the genome assembly for *R. microplus*, we contracted with Dovetail Genomics (Dovetail Genomics, Scotts Valley, CA, USA) to access their chromosome conformation capture Hi-C capability. Using eggs from the Deutsch f12 and f13 generations, Chicago libraries were created *in vitro* by adding synthetic chromatin and crosslinks to facilitate proximity ligation. We also provided Dovetail Genomics with the *R. microplus* polished and assembled genome described above. Data from these libraries and our assembly were analyzed with the Dovetail proprietary algorithm HiRise [9] to find and resolve misjoins in the *de novo* assembly, and to generate the final genome assembly. Genome completeness was assessed using BUSCO v3.0.2 [10] in genome mode with the arthropoda_odb9 BUSCO lineage and the Augustus fly species.

## Ethics Statement

The cattle used to rear the laboratory strains of ticks that provided the eggs for DNA purification were cared for according to protocols approved by the USDA-ARS Cattle Fever Tick Research Laboratory Institutional Animal Care and Use Committee (IACUC).

## Declaration of Competing Interest

This work was funded in parts by the USDA-ARS CRIS Project No. 3094-32000-036-00D, a USDA-ARS Cooperative Agreement No. 58-3094-6-017 with the Department of Entomology, Texas A&M AgriLife Research, College Station, TX, USA, and by Texas A&M AgriLife Research through an Insect Vector Diseases Competitive Grant and High Consequence Genomics Research Project on Vector-borne Diseases to the Department of Entomology. This work used the Extreme Science and Engineering Discovery Environment (XSEDE), which is supported by National Science Foundation grant number ACI-1548562. Specifically, it used the Bridges system, which is supported by NSF award number ACI-1445606, at the Pittsburgh Supercomputing Center (PSC).
